# Antimicrobial resistance in orthopedics: microbial insights, clinical impact, and the necessity of a multidisciplinary approach—a review

**DOI:** 10.2340/17453674.2025.43477

**Published:** 2025-07-23

**Authors:** Julia L VAN AGTMAAL, Mariëlle VERHEUL, Lieve VONKEN, Kato HELSEN, Marian G VARGAS GUERRERO, Sanne W G VAN HOOGSTRATEN, Bianca J HURCK, Giulia PILLA, Isabell TRINH, Gert-Jan DE BRUIJN, Henrik P CALUM, Mark G J DE BOER, Bart G PIJLS, Jacobus J C ARTS

**Affiliations:** 1 Department of Orthopaedic Surgery, Research Institute CAPHRI, Maastricht University Medical Center, Maastricht, the Netherlands; 2 Department of Infectious Diseases, Leiden University Medical Center, Leiden, the Netherlands; 3 Department of Orthopedics, Leiden University Medical Center, Leiden, the Netherlands; 4 Department of Health Promotion, Faculty of Health, Medicine & Life Sciences, Research Institute CAPHRI, Maastricht University, Maastricht, the Netherlands; 5 Department of Communication Studies, University of Antwerp, Antwerp, Belgium; 6 Nostics B. V., Amsterdam, the Netherlands; 7 Department of Clinical Microbiology, Amager and Hvidovre Hospital, University of Copenhagen, Hvidovre, Denmark; 8 Orthopaedic Biomechanics, Department of Biomedical Engineering, Eindhoven University of Technology, Eindhoven, the Netherlands

## Abstract

Antimicrobial resistance (AMR) is rising globally and is a threat and challenge for orthopedic surgery, particularly in managing prosthetic joint infections (PJIs). This review first explores several AMR mechanisms from a microbiological point of view, including selective pressure, horizontal gene transfer, and further dissemination. Second, the variation in the rise of AMR across countries is highlighted, including its impact on PJI. While countries with the highest AMR rates are expected to experience the most significant burden, no country will be immune to the increasing prevalence of PJI. Third, this review stresses that multidimensional strategies are needed to combat AMR’s challenges in orthopedic surgery. These include raising awareness across all sectors, including healthcare professionals, the public, healthcare policymakers, and even politicians; advancing diagnostic technologies for early infection detection and classification of resistant or susceptible strains; promoting antibiotic stewardship; and developing new material technologies to prevent or cure PJI. This review highlights the urgent need for a coordinated response from clinicians, researchers, and policymakers to avoid AMR-related complications in PJI cases.

The discovery of penicillin by Alexander Fleming in 1928 marked the advent of the age of antibiotics. Previously deadly bacterial infectious diseases could be cured in days, revolutionizing medicine. However, over time, the success of antibiotics may be completely cancelled out by their combative counterparts: bacteria that are resistant to many antibiotics commonly used in clinical practice. Antimicrobial resistance (AMR) is the capability of microorganisms (bacteria, viruses, fungi, and parasites) to resist the effects of antimicrobial medicines, particularly antibiotics [1]. AMR greatly complicates and lengthens the treatment of even a simple infection in the human body, dramatically increasing the incidence of further complications or even death. Increased antibiotic-resistant bacteria will result in a tremendous rise in healthcare costs. AMR is a complex, interconnected issue demanding a “one-health” approach that fosters collaboration and surveillance across human, animal, and environmental sectors [2]. The threat of AMR in orthopedics becomes more serious as AMR is rapidly increasing in incidence, and up to 10 million deaths associated with AMR are predicted by 2050 [3-5].

The rise of drug-resistant infections in implant surgeries can have devastating consequences for patient outcomes and treatment efficacy. With aging populations and improved access to healthcare, the global number of total hip and knee arthroplasties (THA and TKA) is rising sharply [6-8]. Despite their overall success, prosthetic joint infection (PJI) follows in 1–4% of these arthroplasties [8-12]. The incidence of infections in open fractures ranges from 30% to 55% [13,14]. Infections result in delayed healing, suboptimal functional outcomes, diminished quality of life, and higher mortality rates [10,15]. Additionally, 30–40% of revision THAs and TKAs result in PJI [8,9,11]. This situation further strains healthcare resources and escalates the economic burden on the system [16]. The treatment of choice for PJI has been a combination of irrigation and surgical debridement to diminish the local bacterial load, coupled with an exchange of implant components with local and systemic antibiotic therapy [17-20]. However, revision is needed again within 25 months for 21% of revised TKAs [20]. Since infections can recur for many years, success rates are measured in survival, i.e., by the number of infection-free survival years without recurrence. As AMR is rising, patients with PJI are at higher risk of therapeutic insufficiency [15,21,22].

First, this article will explore the microbiological mechanisms behind AMR. Second, this study will illustrate the growing threat of AMR and its profound effect on orthopedic surgery. Third, this paper will outline strategies to mitigate AMR, including: raising awareness, advancing diagnostic techniques, promoting antibiotic stewardship, developing new material technologies to prevent or treat PJI, and getting those technologies from bench to bedside.

## Mechanisms of antimicrobial resistance: a microbiological perspective

Throughout evolution, bacteria have survived and evolved through mutation and natural selection, resulting in the cumulative acquisition of various mechanisms to survive threats posed by harmful molecules in their environment. Bacteria isolated from thawed permafrost samples contained different antibiotic-resistance genes and resistance mechanisms, illustrating that AMR development is a natural and ancient phenomenon [23,24]. Bacteria can be intrinsically resistant to an antimicrobial, e.g., all bacteria within a genus share a particular resistance mechanism. Alternatively, bacteria can acquire antimicrobial resistance, resulting in resistance mechanisms that specific strains within a bacterial genus have obtained [25]. Acquired resistance constitutes an increasing worldwide problem driven by the use of antimicrobials, putting selection pressure on bacterial populations. This selective pressure is a strong evolutionary force that causes more resistant bacteria to survive and susceptible bacteria to perish. The development of AMR is inevitable, illustrated by the fact that resistance development has always been observed after the introduction of new antibiotics, or even before their widespread use (Supplementary Figure 1) [26]. Bacteria reproduce rapidly, and the frequency of spontaneous mutations can be extremely high [27]. Mutations with an evolutionary advantage will be passed on vertically to the offspring. In addition, bacteria can exchange DNA on plasmids, including resistance genes, within and between other genera by horizontal gene transfer (HGT) [28]. HGT contributes highly to genome diversity and the spread of acquired AMR within and between bacterial populations. In the one-health perspective framework, antibiotic pollution of the environment by the healthcare, agricultural, and industrial sectors results in antibiotic exposure of bacteria in wastewater, further inducing antibiotic resistance and HGT [29,30].

**Figure 1:**
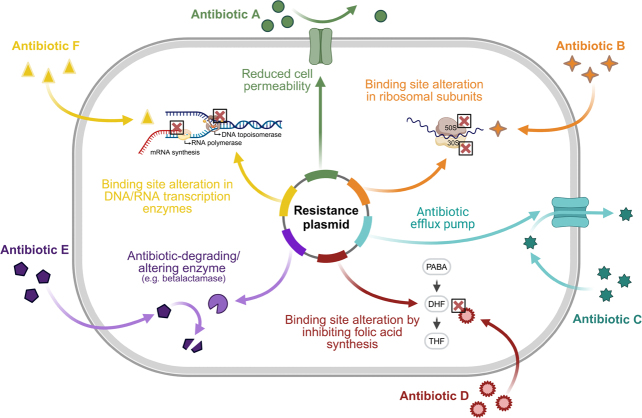
Schematic overview of bacterial resistance mechanisms against the most commonly used antibiotics in clinical practice. Resistance plasmids within bacteria that induce resistance against antibiotics by (A) reducing cell permeability, thereby preventing antibiotics from entering the bacterial cell, (B) binding site alteration in 50s or 30s ribosomal subunits to prevent the binding of antibiotics (lincosamides, tetracyclines) that target these subunits to inhibit protein translation, (C) antibiotic efflux pumps to remove intracellular antibiotics from the bacterial cell, (D) binding site alteration by inhibiting folic acid synthesis, thereby hampering the efficacy of antibiotics that target the folic acid synthesis (trimethoprim, sulfonamides), (E) antibiotic-degrading or -altering enzymes (e.g., betalactamases), and (F) binding site alteration in DNA or RNA transcription enzymes (DNA topo-isomerase, RNA polymerase), thereby inhibiting the efficacy of antibiotics (quinolones, rifamycins) that inhibit the transcription of DNA and RNA. Image created with Biorender.com.

Figure 1 depicts the most common resistance mechanisms in clinically relevant bacterial pathogens. Bacteria can change the structure of the antibiotic target by mutation (Figure 1B, D, and F), e.g., ribosomal subunits or topoisomerase enzymes [31]. Bacteria can break down or modify the antibiotic by the production of hydrolases or antibiotic-degrading enzymes, such as beta-lactamases (Figure 1E) [32,33]. Bacteria can overexpress efflux pumps to remove the antibiotic from the bacterial cell (Figure 1C) [34]. The specificity of these efflux pumps can be narrow and wide, with wide specificity resulting in multidrug resistance efflux pumps. Another resistance mechanism is reducing the cell permeability by decreasing porin expression and permeability to reduce the antibiotic influx (Figure 1A) [35].

Other bacterial mechanisms that aim to thwart the efficacy of antimicrobials should also be considered [36-38]. For example, bacteria can adhere to orthopedic implants and form a biofilm to protect themselves against exogenous stressors, including host immune cells and antibiotics. Bacteria can form biofilms on biotic and abiotic surfaces, and within fluids [39]. The dense biofilm matrix in which the bacteria are embedded consists of polysaccharides, proteins, extracellular DNA, and lipids, hampering the access and efficacy of antimicrobials [40]. Especially in multiple-species biofilms, the biofilm facilitates the exchange of resistance genes by HGT, which enables rapid AMR development [41,42]. Polymicrobial biofilms are particularly abundant ex vivo (e.g., in aqueous environments, on microplastics), but form a vast minority in PJI [43]. In the highly dynamic biofilm system, single bacteria or bacterial aggregates can disperse into the surroundings and potentially form a biofilm elsewhere [44]. Furthermore, biofilm dispersal enables the dissemination of antimicrobial resistance genes obtained within the biofilm, enabling HGT to bacteria outside the biofilm [45]. Importantly, biofilm-embedded bacteria exhibit strategies beyond resistance development, such as antimicrobial tolerance [46]. Bacterial tolerance, often induced by lack of nutrients, hypoxia, and low pH in the biofilm, is characterized by reduced metabolic activity of bacteria and consequently reduced antibiotic target activity [47]. As a result, the time required for complete eradication by antibiotics is increased. Persister cells are a subset of such tolerant cells that are extremely difficult to eradicate by antimicrobial treatment. These antibiotic-tolerant cells persist despite antibiotic exposure and notoriously induce infection relapse after discontinuing antimicrobial treatment. Importantly, antimicrobial tolerance seems to precede and enhance the development of AMR [48]. The ability of bacteria to develop AMR, exchange resistance genes, and further disseminate poses a challenge to our healthcare systems and society.

## AMR influence on PJI risk and orthopedic surgery

**Figure 2:**
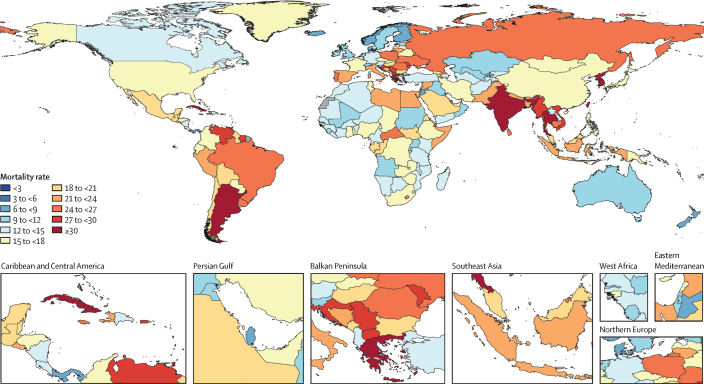
Death rates per 100,000 attributable to AMR, all ages, predicted values for 2050. Reprinted from Naghavi et al. (2024) [5], with permission according to the Creative Commons CC-BY license https://creativecommons.org/licenses/by/4.0/.

AMR is among the top 10 global public health threats, as declared by the World Health Organization (WHO) [4,49]. By 2050, AMR is expected to be associated with up to 10 million deaths [3-5,50]. Antibiotic-resistant bacteria will increase infection rates and worsen treatment results in most surgical interventions, cancer treatments, and potentially other diseases as well [50,51]. A high heterogeneity in AMR mortality rate is expected per country (Figure 2), with the highest estimates in low- and middle-income countries like Africa, South Asia, Latin America, and the Caribbean [5]. These incidence (and cost) numbers highlight the critical need for a multipronged approach to tackling AMR.

**Figure 3:**
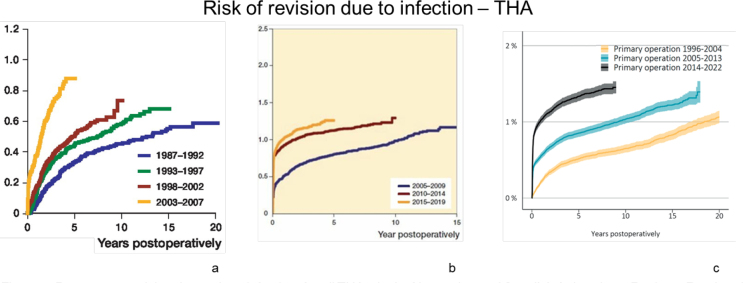
Percentage revision due to deep infection, for all THAs, in the Norwegian and Swedish Arthroplasty Register. Reprinted from (A) Dale et al. (2009) [62], (B) Dale et al. (2021) [63], and (C) the Swedish Arthroplasty Register (2023) [64] (CRR = cumulative risk of revision due to infection) with permission according to the Creative Commons CC-BY license https://creativecommons.org/licenses/by/4.0/.

This discrepancy between countries is also observed in reported PJI rates that vary considerably between countries, between 0.43% and 4.73% for THA and 1.52% and 2.94% for TKA [52-59]. However, the study period and length of the follow-up, as well as reporting bias, might affect the results, complicating comparisons across studies. Though the current percentage of PJIs is relatively low in Nordic countries, the Netherlands, Wales, and the UK, the incidence is rising. The low incidence might be due to accurate tracking of infection numbers, leading to improved surveillance and prevention, and enhanced and proactive treatment protocols [57-61]. Also, when diagnostic tools become more specific and accurate, infection incidence might be lowered because false positives are omitted. On the other hand, the selection criteria for total joint replacement have widened in the last two decades, and patients with more comorbidities that can result in a higher infection incidence are more commonly operated on. Dale et al. [62,63] reported an increase in the percentage of THA revisions due to PJI from 1987 to 2019 (Figures 3A and B). From 1987 to 2007, the risk of PJI increased threefold [62], and from 2005 to 2019, it again increased [63]. Likewise, in Sweden (Figure 3C), the risk of revision due to infection has been growing over the years, both shortly after surgery and several years postoperatively [64].

Kamp et al. [65] found a mismatch in the total PJIs in a regional infection cohort (1% acute PJI incidence) compared with the Landelijke Registratie Orthopedische Interventies (LROI) data (0.6% acute PJI incidence), as debridement antibiotics and implant retention (DAIR) procedures are not included in the LROI’s PJI number. Furthermore, PJIs were missing for administrative reasons. These numbers base the prevalence of PJIs on the number of revisions needed. Currently, most infections are stopped by antibiotics. With the rise of AMR, these infections might progress due to therapeutic insufficiency.

Although there has been a rise in multidrug-resistant PJIs (e.g., 9.3% to 15.8% from 2003 to 2012 [66]), the number of PJIs caused by (multi)drug-resistant PJIs is scarce [66-68]. These antibiotic-resistant bacteria will also be harder to eradicate. Therefore, modifying treatment algorithms—especially shifting from systemic to local antibiotic treatment, enabling higher antibiotic dosing—could help control the increase in infection incidence due to AMR. Also, a change from intravenous to oral antibiotics is ongoing to shift expensive hospital care towards the home environment [69]. Moreover, pre-, peri-, and postoperative infection prevention remains crucial, including surgical skin preparation, prophylactic antibiotics, nutritional status, weight optimization, smoking cessation, decolonization of nasal cavity bacteria, and hand and operating room hygiene [70,71]. The higher infection incidence and more antibiotic-resistant bacteria can result in higher treatment failure rates [68]. Maintaining the currently low infection rates in primary joint arthroplasty seems unlikely in the coming decades, leading to more revision surgeries and high associated healthcare costs.

## Mitigating and preventing AMR in orthopedics

AMR is a wicked problem that requires action across various sectors, including healthcare, where elements within and between different settings interact. Several other sectors also contribute to the exacerbation of AMR. The improper use of antibiotics in intensive livestock farming, inadequate wastewater treatment, and increased global travel worsen AMR. Therefore, stakeholders from these sectors must play a role in addressing it. This chapter focuses on tackling AMR within orthopedics by examining key aspects, including raising awareness and promoting behavior change among healthcare professionals (HCPs) and the public, advancements in diagnostics, the development of new material technologies, and the challenges of clinical implementation. Awareness and improved diagnostics are required to prevent AMR. Clinically, optimization of early infection diagnostics is essential to improve infection treatment and prevent AMR occurrence. While collaboration extends beyond stakeholders directly involved in these areas, interdisciplinary efforts are crucial.

DARTBAC is an interdisciplinary Dutch consortium that unites academia, industry, and government to mitigate AMR. This multidisciplinary consortium comprises 26 partners with expertise in infection diagnostics and treatment, microbiology, material technology, clinical and molecular imaging, and social sciences, including methods for achieving behavior change. All partners are focused on enhancing AMR awareness and developing new material technology solutions that do not rely solely on antibiotics to combat infections and antibiotic-resistant bacteria.

### Awareness, behavior change, and antibiotic stewardship

**Figure 4:**
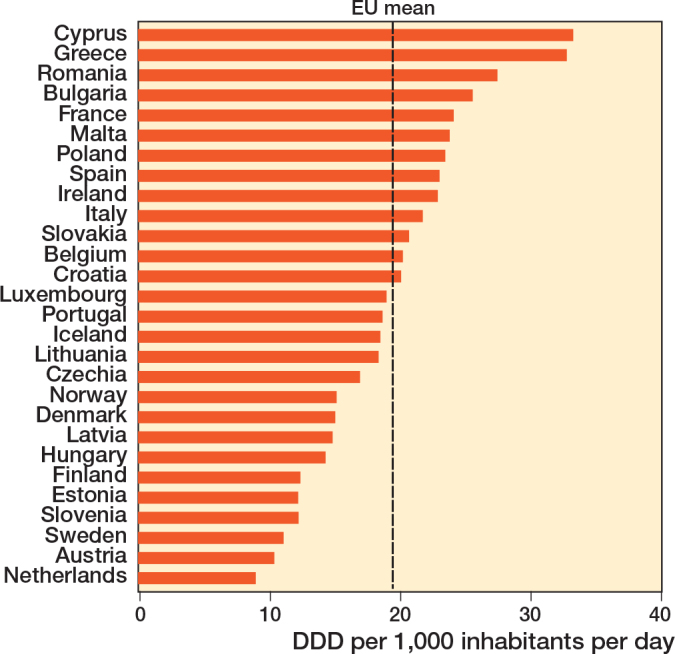
Data from the European Centre for Disease Prevention and Control (ECDC) surveillance report on total consumption (community and hospital sector) of antibacterials for systemic use in 2022, expressed as Defined Daily Dose (DDD) per 1,000 inhabitants per day (European Centre for Disease Prevention and Control, 2022) [73].

Awareness, behavior, and antibiotic stewardship are necessary components in the initiatives to curb AMR [72]. Accordingly, the primary goal of antibiotic stewardship is better patient care. As is often mistakenly assumed, the goal is not to reduce antibiotic use or save costs. However, they can be considered favorable secondary outcomes. Therefore, the EU has set a goal to reduce antibiotic use by 20% by 2030 compared with the baseline year 2019. The European Centre for Disease Prevention and Control (ECDC) has measured a 2.5% reduction in 2022 (Figure 4) [73]. Nearly half of the EU Member States saw increased antibiotic consumption between 2019 and 2022, highlighting the need for intensified action to meet the EU’s goals [73]. Policy initiatives acknowledge the overuse and misuse of antimicrobials as the main driver for resistance development, and the need to optimize antimicrobial use [74]. Although there is an EU goal to reduce antibiotic prescription and use, no general guidelines exist to reach this goal. European guidelines, such as those developed to prevent, diagnose, and treat fracture-related infections, would be beneficial for a standardized approach [75].

Following this, the focus for stewardship efforts should be on optimizing appropriate antibiotic use and promoting the use of the right antimicrobial agent at the correct dosage and for the proper duration [76]. Many countries have implemented successful National Action Plans on antimicrobial resistance, in which antibiotic stewardship is a key component and a priority. Although some policies have demonstrated clear benefits in reducing antimicrobial misuse, comprehensive evaluations of these successful policies are often lacking. There is limited information on critical aspects like cost-effectiveness, and inadequate descriptions of the technical and regulatory frameworks required for implementation and necessary regulatory changes [77]. Moreover, insights from behavior change research, including evidence-based behavior change strategies, are applied insufficiently [78].

Although healthcare professionals concur that AMR is a global issue, they often do not perceive it as a serious local problem [79,80]. Communication strategies emphasizing the closeness and concreteness of AMR are required to change these perceptions. These strategies should consider the complex situation faced by HCPs, where incentive structures, complex networks of decision-makers, and complex choices and outcomes complicate responsible AMR-related behavior [81]. For example, antibiotic prescribing creates a complex social dilemma. HCPs often prioritize the immediate safety of the patient by (over)prescribing antibiotics, despite the long-term negative consequences of increased AMR at the population level [81]. Theory-based approaches for managing social dilemmas should be applied to counteract this effect [82].

The main challenge currently is to implement stewardship in communities. While antimicrobial stewardship is developing rapidly at the hospital level, it requires significantly more attention and development at the community level [83]. As both users of antibiotics and potential contributors to the spread of AMR, the general public play an essential role in curbing AMR [84,85]. However, the general public often has a limited understanding of AMR, resulting in misconceptions and risky behaviors, such as not following medical prescriptions or requesting unnecessary antibiotics [86-88]. The general public’s awareness and knowledge related to AMR and their understanding of the causes and consequences of AMR should be enhanced [87,89]. However, a large amount of the population is not reached by awareness campaigns, contributing to the discrepancy in knowledge and potentially driving irrational antibiotic use [90]. In addition, the complex terminology related to AMR hampers remembrance and accurate risk perception [91]. Clear communication strategies targeted at the general public are essential to fostering a more informed and proactive approach to combating AMR.

The widespread perception that AMR is a distant and abstract problem might reduce the willingness of all stakeholders to act against AMR. Traditional communication strategies to raise general awareness are unsuitable for resolving this misconception. Effective communication can only be developed when the system surrounding stakeholders is fully understood, allowing relevant intervention points to be identified. Promoting behavior change requires a combination of awareness, motivation, and a supporting (social) environment [92]. While awareness is a prerequisite for behavior change, other behavioral determinants such as skills and social influences are equally important and warrant further research [84,93]. Understanding which determinants need to be changed enables adequate selection of evidence-based behavior change strategies, such as education, incentivization, and providing appropriate role models [94].

### Diagnostic development

PJIs present with variable clinical symptoms depending on the patient and infection; no conclusive diagnostic method is available to confirm the presence of an infection and identify the causative pathogens and their antimicrobial susceptibility. Often, a combination of sample collection methods, diagnostic techniques, culture-based methods, imaging, and molecular analysis is required [95]. Although progress has been made towards diagnostic methods for PJIs, challenges remain in achieving faster diagnosis and enhancing sensitivity and specificity. Current phenotypic antimicrobial susceptibility testing (AST) is still culture-based and provides results only after 18–24 hours [96]. Clinical microbiological laboratories possess various AST testing systems to guide antibiotic treatment. However, these (semi-)automated systems cannot detect bacterial tolerance or predict AMR development and are slow to report antimicrobial susceptibility [97]. New testing technologies aim to improve treatment by enabling rapid detection and identification of causative bacteria and their antibiotic susceptibility.

Different types of sensors are being developed for bacterial detection and AST. Nano-mechanical detection (nano-motion) and heat detection are promising options, with the latter being particularly interesting for detecting PJIs [98,99]. CRISPR-Cas-based biosensing applications can detect genetic material and present a low-cost, easy-to-use option with high specificity and sensitivity [100,101]. However, while effective for virus detection, it shares the same limitations as traditional molecular methods when applied to bacteria. Bacteriophage (phage)-based detection and species differentiation use reporter phage-induced bioluminescence and can identify live infections at an early stage [102,103]. Despite this advantage, scalability remains a challenge due to the complexity of phage engineering and regulatory hurdles. Microscopy-based technologies have also made significant progress [104]. Monitoring bacterial growth using automated time-lapse microscopy or photomicrography allows the detection of single-cell morphological changes using bright-field microscopy. Costs, particularly for equipment and maintenance, still limit this type of technology; however, it has the advantage of detecting viable pathogens.

Finally, label-free, spectroscopic methods such as Fourier Transform Infrared (FTIR) spectroscopy and Surface-enhanced Raman Spectroscopy (SERS) are promising technologies due to their versatility and cost-effectiveness [105-107]. As different bacterial phenotypes have distinct signatures due to their composition and metabolism, these spectroscopic methods enable label-free detection and identification of viable bacteria and potentially AST. These technologies provide rapid results and can be applied directly to samples without requiring cultures, while maintaining high sensitivity and accuracy. Advancements in machine learning methods are expected to result in the automation of routine procedures such as microbial cytopathology, microscopy analysis, colony counting, and culture-based AST, leading to significant improvements in accuracy and diagnosis speed [107].

### New materials, technologies, and innovative approaches for PJI prevention and treatment

A broad range of material technologies with antimicrobial properties is being developed to counter the emergence and spread of AMR. Traditionally, antimicrobial technologies have been divided into bacteriostatic or bactericidal classes. Bacteriostatic agents inhibit bacterial growth and reproduction by inhibiting protein or folate synthesis, DNA synthesis or replication, or other metabolic functions [108,109]. Bactericidal agents eradicate the bacteria by disrupting vital cellular processes or structures, such as DNA fragmentation, inhibiting cell wall synthesis, and membrane integrity [108,100]. Antimicrobial agents often exhibit both effects, depending on the concentration, bacterial species, and other (test) conditions [108].

#### Active peptide compounds

Antimicrobial peptides (AMPs) exhibit broad-spectrum antimicrobial activity against bacteria, viruses, and fungi, and are naturally occurring in almost all life forms, or can be synthesized [110]. These peptides primarily target and disrupt bacterial cell membranes through hydrophobic or electrostatic interactions, causing lysis of the cell [97]. While there are already a few FDA-approved AMPs for wound infection treatment, challenges regarding stability, antibacterial efficacy, and environmental sensitivity remain present [111]. Synthetic antimicrobial and antibiofilm peptides (SAAPs) are synthetic versions of natural AMPs, which can more effectively target and disrupt bacterial membranes and penetrate bacterial cells to reach intracellular targets. SAAP-148 demonstrated a bactericidal effect against antibiotic-resistant pathogens, without inducing bacterial resistance upon long-term exposure [112]. However, protein binding can reduce bioavailability, lowering antimicrobial activity [113]. Encapsulation of AMPs may preserve their antimicrobial activity and reduce cytotoxicity [114]. Bioavailability should be increased while finding an effective yet safe dosage before moving to clinical trials.

#### Bacteriophage treatment

Difficult-to-treat infections and the global spread of multidrug-resistant bacteria have reignited interest in bacteriophage (phage) therapy. Phages are the natural predators of bacteria and are highly diverse, ubiquitous, and abundant on Earth [115]. These viruses can infect and kill a specific bacterial species or bacterial strain(s) within a species. Phages recognize a specific surface receptor or several receptors on the bacterial cell wall, e.g., polysaccharides and peptide sequences, which can be highly diverse between bacterial strains [116]. After attachment to a susceptible bacterium, lytic phages insert their genome into the cytoplasm and hijack the bacterial replication and translation machinery to ensure viral reproduction. This results in bacterial lysis, cell death, and the release of the phage progeny. For therapeutic purposes, selecting a phage suitable for the specific bacterial strain infecting the patient is crucial due to the phage’s specificity. Further, phages can be pre-adapted to bacteria by co-evolution to broaden the phage’s host range, enhance bacterial eradication, and reduce the development of phage-resistant bacteria [117,118]. Phage therapy, especially when combined with antibiotics, resulted in the elimination of most of the bacterial infections in PJI [119]. Patients with PJI receiving phage therapy during surgical debridement did not show infection recurrence [120]. Generally, the incidence of adverse events due to phage therapy was low, and the adverse events that have been reported were considered mild and resolvable [119]. Clinical phage trials using predefined phage cocktails could not reproduce the positive treatment outcomes observed in case reports. Hence, personalized approaches for phage therapy would be more suitable for clinical phage trials, as recently reported in combination with standard-of-care antibiotics [121,122].

Phage therapy for bone and joint infections lacks a standardized treatment protocol, for which high-quality clinical trials are required [123]. Because phage therapy is still considered experimental in Western Europe, it is restricted to “last resort” options for patients who have undergone extensive treatments. Altogether, a high-throughput system is needed for personalized phage treatment, and regulations must be adjusted to ensure rapid phage selection and administration while evaluating the safety, quality, and efficacy of phage therapy [122].

#### Bioactive glass

Bioactive glasses (BG) are a group of surface-reactive glass-ceramic biomaterials. When bioactive glass is implanted into the body, it will react with the surrounding bodily fluids. This reaction involves the exchange of ions within the glass with hydrogen ions from the fluids, creating an alkaline microenvironment due to a pH increase. In addition, the ion release from BG increases the osmotic pressure. These combined effects effectively inhibit bacterial growth and result in a mechanical attack on the bacterial cell wall [124]. Studies have found that S53P4 bioactive glass granules are very effective against many bacterial strains, such as methicillin-resistant *Staphylococcus aureus* [125]. Clinical results in osteomyelitis are excellent, with eradication rates above 90% in one-stage treatment [126,127]. Although S53P4 bioactive glass granules are efficient in osteomyelitis treatment, the granular form is inadequate for implant protection. Therefore, new formulations are being developed. S53P4 nanoparticulate powder, for instance, exhibits more pronounced effects on environmental pH and osmolarity changes [128,129]. Therefore, the antibacterial effect also occurs faster compared with the granules, as the ions are readily available [129]. BG could potentially be used for implant protection by preventing bacterial adherence or biofilm formation or by eradicating these issues [129].

#### Bioceramics

Bioceramics, like ion-substituted calcium phosphate, usually comprise hydroxyapatite, tricalcium phosphate, or a combination of both compounds. They can be formulated as granules or cement and are considered osteoconductive and bioactive [130-132]. In infection treatment, several material classes can be identified: bioceramic calcium sulphate, calcium phosphate materials, combinations of the two, and ion-substituted calcium phosphate materials. Bioceramics are usually mixed with antibiotics and can be used to reconstruct bone defects after infection eradication. The composition ratio of calcium phosphate and calcium sulphate affects the material’s mechanical strength, resorption rate, and pharmacokinetic release of embedded antibiotics [133]. These materials have reported clinically effective infection eradication results of over 90% in osteomyelitis patient cohorts [134,135]. However, due to antibiotic dependence, bioceramics are susceptible to AMR development. Ion-substituted calcium phosphates can be combined with other antimicrobial compounds such as selenite, copper, zinc, rubidium, gadolinium, silver, and samarium [136]. These combination biomaterials have demonstrated antimicrobial effects in vitro and in vivo; however, they have not yet reached clinical implementation.

#### Induction heating

Non-contact induction heating (NCIH) is a non-invasive treatment modality that can potentially be used to cause thermal damage to the bacteria within the biofilm on the metal implant surface [137-139]. NCIH uses pulsed electromagnetic fields (PEMFs) to induce so-called “eddy currents” within metal objects, which causes them to heat [137-139]. NCIH typically uses frequencies between 10 kHz and 500 kHz, which actively heats only the metal implant and has no direct heating effect on the surrounding tissue [137-140]. In addition to non-invasive use, NCIH could also be applied during surgery of an infected implant to increase the effectiveness of, e.g., DAIR [137,140]. NCIH can, for instance, heat parts of the implant that cannot be easily reached (e.g., posterior femoral condyles) or that are very difficult to clean [141]. Several in vitro studies have shown a reduced bacterial load due to the NCIH on metal implants, with some even demonstrating complete eradication of mature biofilms and others showing a synergistic effect with other antimicrobial compounds [137,142-144]. Progression to in vivo studies is being conducted [145]. Interestingly, persister cells within mature biofilms were highly susceptible to NCIH [143]. A recent study has suggested that NCIH increased the susceptibility of meropenem-resistant Pseudomonas aeruginosa to meropenem treatment [142]. In conclusion, NCIH of metal implants could play an important future role in the multimodality treatment of PJI combined with other therapies [146].

#### Metal-based material technologies

Metal(-based) material technologies, including metal ions, nanoparticles, and complexes, are gaining attention as potential antimicrobial agents [147]. Their efficacy and stability vary with structure, such as salts, alloys, and nanoparticles, and depend on the application [148]. Metals such as silver, copper, and zinc have long been known for their antimicrobial properties and have been used in various medical applications. The main antimicrobial mechanisms of these metals are cell membrane disruption and the generation of reactive oxygen species, which interfere with essential cellular processes [149]. Some silver-based coatings are available in the clinic; however, the use of these coated implants is only justified in high-risk cases, as there is a lack of prospective randomized clinical trials [150]. While there are some challenges regarding understanding the underlying antibacterial mechanism of metals and their impact on the host immunity system, the use of metals holds great potential to combat antimicrobial resistance [151,152].

#### Surface topography modifications

The primary working mechanism of bactericidal surfaces is either chemical or physical. Common chemical methods use surface bio-functionalization or surface coatings to enhance the antibacterial properties of the surface [153]. However, after repeated exposure, some bacterial strains develop resistance. This important limitation underscores the importance of physical mechanisms to combat implant-associated infections. By producing specific nanopatterns in implant surfaces, cell fate can be influenced [153]. Nanopatterns can be varied in shapes like nanopillars and nano grooves, and they can be varied in size parameters such as height, width, depth, and spacing. Subsequently, the size modulates the interaction of nanopatterns with cells. Many studies have shown that high-aspect ratio nanopatterns are capable of killing bacteria [153,154], preventing bacterial adhesion [155-157], and prevention of biofilm formation [155].

#### Polymers

Polymers are promising materials in the antimicrobial research field [158]. Their properties—like molecular weight, functional groups, and hydrophobicity—can easily be tuned to fit the intended application [159]. Some polymers have intrinsic antimicrobial properties (e.g., chitin [160], and chitosan [161]), for others, their functional groups can be modified to be antimicrobial (e.g., quaternary ammonium compounds [159]), and some polymers contain antimicrobial compounds (e.g., silver [162]). Polymers with antimicrobial properties are divided into passive (repelling bacteria) and active (killing bacteria) [163]. Passive polymers are either hydrophilic, negatively charged, possess low surface energy, or a combination of those properties [163,164]. Active polymers are usually functionalized with antimicrobial compounds like antibiotics, peptides, or cations (quaternary ammonium compounds) [159,163]. An example of an active compound is the polymer coating loaded with the peptide chicken cathelicidin-2, exhibiting strong antibacterial activity for 4 days [165]. Bacteria are less prone to develop resistance against antimicrobial polymers compared with antibiotics due to their many unique antimicrobial mechanisms [159]. These mechanisms include: inflicting physical damage to the bacteria (especially cationic polymers, which are known for disrupting the cell membrane [166]), oxidative stress [167,168], and surface modification to kill or prevent bacteria from settling on the polymer surface [169].

### Future outlook

Numerous antimicrobial agents are being explored, which are not covered in this summary, e.g., biofilm enzyme inhibitors, quorum-sensing inhibitors, and plant-based substances [118,170-172]. A major challenge in developing new antimicrobial technologies is achieving a balance between their antimicrobial properties and biocompatibility, which must be carefully considered during the development process [173]. The optimization of these active compounds holds great potential for addressing the growing challenge of antimicrobial resistance, which gives hope in the ongoing battle against AMR.

### From bench to bedside—barriers to clinical implementation of emerging technologies

Though multiple new technologies are being developed to counter AMR, few of these technologies have reached clinical implementation due to the difficulty in translating results from in vitro to in vivo to clinical tests. The standard test methods that are currently available are not specifically for medical products. Furthermore, as new material technologies have different working mechanisms to counter AMR, the in vitro tests also vary, and comparing results and setting minimal requirements to proceed to in vivo experiments is difficult [174].

Just as no in vitro model is ideal, no in vivo model can fully replicate all aspects of the human biological environment. Once an antibacterial technology proves effective in vivo for preventing PJI, the clinical efficacy can be evaluated through randomized controlled trials. However, these trials may have limitations in predicting effectiveness, including the risk of incorrect statistical inference [175,176].

Conflicting demands from different stakeholders hinder the translation of experimental antimicrobial surface designs from research to clinical use; the interplay between researchers, industry, insurers, policymakers, payers, and regulatory agencies complicates translation. Despite the recognized need for improved antimicrobial technologies, the risks associated with the translation process often outweigh the potential benefits, resulting in many promising designs failing to reach clinical application [177]. Most of these designs fail in testing or never progress to in vivo experiments due to financial and industrial limitations [178]. The patient population and market opportunities are relatively small, despite high development costs.

The regulatory procedures demand clinical validation levels that are statistically and financially unrealistic to meet, especially with a small patient population [177]. This is often the point where development halts due to costs and feasibility [178]. Both European acceptance by the MDR and the USA FDA require these expensive trials, even when individual components are already validated, or when trial data is available from the other regulatory bodies [177]. Despite these challenges, there is a strong need for new antimicrobial innovations to address the limitations of existing infection prevention measures. To develop better strategies to assess antimicrobial techniques without relying solely on costly clinical trials, the cooperation of all stakeholders is needed.

### Conclusions

Antimicrobial resistance (AMR) is predicted to be associated with 10 million annual deaths by 2050 if left unchecked. Therefore, the World Health Assembly’s 2015 Global Action Plan on AMR and the 2017 UN General Assembly declaration both acknowledge AMR as a global public health threat. The ability of bacteria to develop AMR, exchange resistance genes, and further disseminate poses a challenge to our healthcare systems and society. With AMR on the rise, it poses significant challenges to effective PJI management. In addition to increasing difficulty in treating PJI, the incidence of PJI is rising even in high-income countries with improved surgical and implant techniques.

Multidimensional strategies are needed to combat AMR’s challenges in orthopedic surgery. First, AMR awareness among all stakeholders is a prerequisite for behavior change. The public and healthcare professionals must understand the threat of AMR and which individual actions they can take. Moreover, among others, the pharmaceutical industry, farmers, veterinarians, politicians, and policymakers should be included in AMR-combating strategies. AMR awareness should lead to sound antibiotic stewardship. Antibiotic stewardship should focus on ensuring the proper use of antibiotics by selecting the right antimicrobial agent, administering it at the correct dosage, and for the appropriate duration. This stewardship is not limited to the hospital level but transcends to the community level. Second, no definitive diagnostic method exists to confirm an infection and identify the causative pathogens. Machine learning methods are expected to be the future of diagnostics, resulting in the automation of microbial cytopathology, microscopy analysis, colony counting, and culture-based AST. This is expected to improve time-to-diagnosis, sensitivity, and specificity. Third, the emergence and spread of AMR require the development of novel therapeutic strategies. A broad range of material technologies with antimicrobial properties is being developed. Moreover, new antibiotics are desperately needed. Due to the difficulty in translating results from in vitro to in vivo to clinical tests, few new antimicrobial technologies have yet reached clinical implementation.

### Funding, use of AI, and disclosures

This publication is part of the DARTBAC project (with project number NWA.1292.19.354) of the research program NWA-ORC, which is (partly) financed by the Dutch Research Council (NWO). Complete disclosure of interest forms according to ICMJE are available on the article page, doi: 10.2340/17453674.2025.43477

### Supplementary data

A Supplementary Figure is available as supplementary data on the article page, doi: 10.2340/17453674.2025.43477

## Supplementary Material



## Notes

JLvA and JJCA: conceptualization and project management; all authors: contributed in their specific field of expertise and reviewed the article; JLvA: edited the last version; JJCA: supervised. All authors have read and agreed to the published version of the manuscript.

Handling co-editors: Marianne Westberg and Philippe Wagner

*Acta* thanks Kaisa Huotari and other anonymous reviewers for help with peer review of this manuscript
