# Supplementary material for: Antimicrobial resistance in orthopedics: microbial insights, clinical impact, and the necessity of a multidisciplinary approach—a review
Source: Acta Orthop. 2025 Jul 23;94:555–68. doi: 10.2340/17453674.2025.43477 (PMC12285513; doi:10.2340/17453674.2025.43477)
Supplement: Supplementary file 1 [file ActaO-96-43477-s1.pdf]

Supplementary Figure

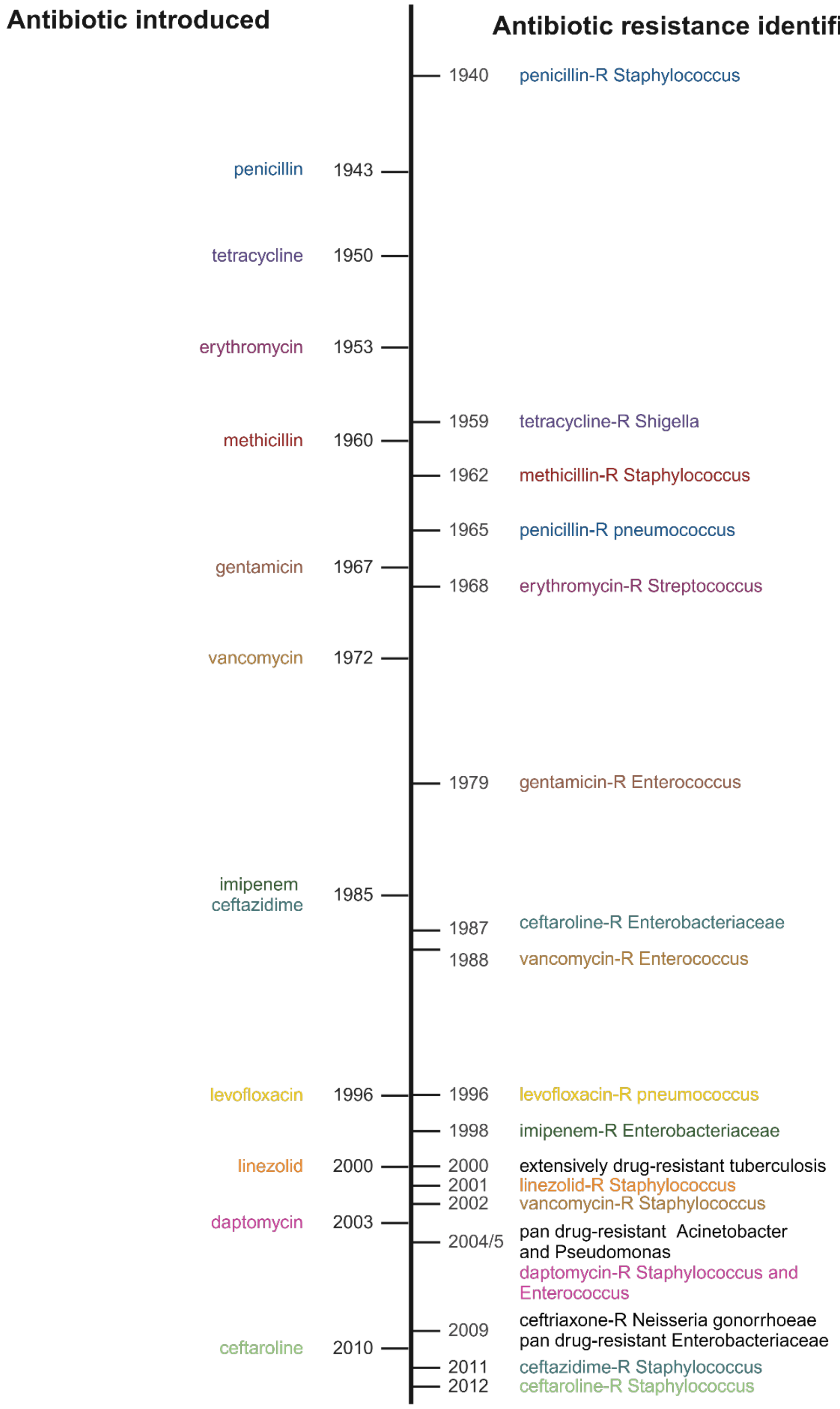

Supplementary Figure 1. Timeline of introduction of antibiotics, paired in colour with the first reported resistance of these antibiotics. Cited from the Centers for Disease Control and Prevention: Dates are based upon early reports of resistance in the literature [179]. Daptomycin resistance [180] and ceftaroline resistance [181] are added to the CDC data. Created with Biorender.com
